# Evidence for a causal association between milk intake and cardiometabolic disease outcomes using a two-sample Mendelian Randomization analysis in up to 1,904,220 individuals

**DOI:** 10.1038/s41366-021-00841-2

**Published:** 2021-05-24

**Authors:** Karani Santhanakrishnan Vimaleswaran, Ang Zhou, Alana Cavadino, Elina Hyppönen

**Affiliations:** 1 Hugh Sinclair Unit of Human Nutrition, University of Reading, Reading, UK; 2 Population, Policy and Practice, UCL Institute of Child Health, London, UK; 3 Institute for Food, Nutrition, and Health, University of Reading, Reading, UK; 4 Australian Centre for Precision Health, Unit of Clinical and Health Sciences, University of South Australia, Adelaide, SA Australia; 5 Section of Epidemiology and Biostatistics, School of Population Health, University of Auckland, Auckland, New Zealand; 6 South Australian Health and Medical Research Institute, Adelaide, SA Australia

**Keywords:** Nutrition, Nutrition disorders, Epidemiology

## Abstract

**Background:**

High milk intake has been associated with cardio-metabolic risk. We conducted a Mendelian Randomization (MR) study to obtain evidence for the causal relationship between milk consumption and cardio-metabolic traits using the lactase persistence (*LCT*-13910 C > T, rs4988235) variant as an instrumental variable.

**Methods:**

We tested the association of *LCT* genotype with milk consumption (for validation) and with cardio-metabolic traits (for a possible causal association) in a meta-analysis of the data from three large-scale population-based studies (1958 British Birth Cohort, Health and Retirement study, and UK Biobank) with up to 417,236 participants and using summary statistics from consortia meta-analyses on intermediate traits (*N* = 123,665–697,307) and extended to cover disease endpoints (*N* = 86,995–149,821).

**Results:**

In the UK Biobank, carriers of ‘T’ allele of *LCT* variant were more likely to consume milk (*P* = 7.02 × 10^−14^). In meta-analysis including UK Biobank, the 1958BC, the HRS, and consortia-based studies, under an additive model, ‘T’ allele was associated with higher body mass index (BMI) (*P*_meta-analysis_ = 4.68 × 10^−12^) and lower total cholesterol (TC) (*P* = 2.40 × 10^−36^), low-density lipoprotein cholesterol (LDL-C) (*P* = 2.08 × 10^−26^) and high-density lipoprotein cholesterol (HDL-C) (*P* = 9.40 × 10^−13^). In consortia meta-analyses, ‘T’ allele was associated with a lower risk of coronary artery disease (OR:0.86, 95% CI:0.75–0.99) but not with type 2 diabetes (OR:1.06, 95% CI:0.97–1.16). Furthermore, the two-sample MR analysis showed a causal association between genetically instrumented milk intake and higher BMI (*P* = 3.60 × 10^−5^) and body fat (total body fat, leg fat, arm fat and trunk fat; *P* < 1.37 × 10^−6^) and lower LDL-C (*P* = 3.60 × 10^−6^), TC (*P* = 1.90 × 10^−6^) and HDL-C (*P* = 3.00 × 10^−5^).

**Conclusions:**

Our large-scale MR study provides genetic evidence for the association of milk consumption with higher BMI but lower serum cholesterol levels. These data suggest no need to limit milk intakes with respect to cardiovascular disease risk, with the suggested benefits requiring confirmation in further studies.

## Background

Obesity, hypertension, dyslipidaemias and hyperglycaemia are all strong contributors’ to cardio-metabolic diseases, which are the major causes of morbidity and mortality worldwide [1–3]. Diet is a major determinant of cardio-metabolic diseases [4] and several studies have shown associations between dairy and milk consumption and cardio-metabolic risk factors [5–10]. High fat dairy products can lead to adverse effects on cardiovascular risk by increasing the intake of saturated fat and cholesterol which have been shown to increase blood cholesterol and subsequent risk of cardiovascular diseases (CVDs) [11, 12]. In addition, milk is a major source of calcium and a risk factor for arterial calcification [13]. Despite these proposed adverse effects, a reduced risk of CVDs was reported for consumption of milk and low-fat dairy products in large scale meta-analysis of data from nine studies (*N* = 57,256) [8]. The findings from randomised controlled trials (RCTs) have been inconsistent [14], failing to provide causal evidence either for a beneficial or adverse causal association.

The lactase enzyme, which is responsible for the digestion of milk sugar lactose, is encoded by the lactase (*LCT*) gene. A functional single nucleotide polymorphism (SNP), −13910 C/T (rs4988235), located upstream of the *LCT* gene, has been shown to affect the transcription of the enzyme and control the distribution of lactase phenotypes in human populations. The ‘T’ allele of the SNP rs4988235 is associated with lactase persistence (LP) and has been shown to increase the *LCT* gene promoter activity after binding transcription factors [15]. Given the functional impact of this SNP on the digestion of milk sugar lactose, the SNP rs4988235 has been used as proxy for milk intake in Mendelian randomization (MR) studies that examined the relationship between milk intake and diseases [16–18].

Recent studies have demonstrated a causal relationship between higher dairy intake and higher body mass index (BMI) [19, 20] but not CVD-related outcomes [21]. Given the discrepancies, we set out to comprehensively examine the causal association between milk consumption and CVD, Type 2 Diabetes (T2D) and cardio-metabolic risk factors, including measures of adiposity, blood pressure, and markers of chronic inflammation, lipid and glucose metabolism. Our MR study included meta-analysis of the data in up to 417,236 individuals from three large population-based studies [22–24], and summary statistics data from several large consortia meta-analyses (*N* up to 1,486,984) [25–29].

## Methods

### Study populations

The full details of the three study populations [the 1958 British Birth cohort (1958BC), Health Retirement Study (HRS) and UK Biobank] are included in the Supplementary file. Briefly, for the present study, we have included up to 5672 individuals from the 1958BC, 8520 participants from the HRS and 404,648 individuals from UK Biobank.

### Biomarker information and disease classification

Details on anthropometric data and biomarkers available from each of the studies are provided in Supplementary Table 1 and laboratory procedures are detailed in Supplementary methods. For the HRS, details of biomarkers are provided in the HRS documentation (http://hrsonline.isr.umich.edu/modules/meta/bio2008/desc/Biomarker2006and2008.pdf). For the UK Biobank, details of anthropometric and biochemical parameters can be found in the following links: https://biobank.ndph.ox.ac.uk/ukb/ukb/docs/Anthropometry.pdf and https://biobank.ndph.ox.ac.uk/showcase/refer.cgi?id=1227, respectively. In the UK Biobank, T2D cases were identified using the relevant first occurrence data fields (Supplementary file), which were generated by combining information from primary care data, hospital inpatient data, mortality data and self-reported medical conditions reported at the baseline or follow-ups. CVD events were ascertained from the hospital inpatient data and mortality data, and were identified using ICD codes (Supplementary file).

### Milk intake

Information on milk intake was available from the 1958BC and the UK Biobank. For the 1958BC, milk intake variable was derived from the 45 years biomedical survey and based on the question “how often do you drink milk alone or in milky drinks”. In the UK Biobank, milk intake was derived from a question “What type of milk do you mainly use?” included in the touchscreen questionnaire (http://biobank.ctsu.ox.ac.uk/crystal/refer.cgi?id=100312).

### Genotyping and SNP selection

SNP rs4988235 was genotyped in all three cohorts. For 1958BC, this SNP was derived from genome-wide association study (GWAS) data (*N* = 2500 from Illumina Infinium 550 K chip, *N* = 3000 from Affymetrix 6.0 platform). For HRS, the DNA samples were genotyped at the Center for Inherited Disease Research using the Illumina HumanOmni2.5-4v1 array and using the calling algorithm GenomeStudio version 2011.2, Genotyping Module 1.9.4 and GenTrain version 1.0. For the UK Biobank, samples were genotyped at the Affymetrix Research Services Laboratory in Santa Clara, California, USA. Axiom Array plates were processed on the Affymetrix GeneTitan^®^ Multi-Channel (MC) Instrument. Further details of DNA analysis can be found in the Supplementary file.

### Consortia-based studies

We have also used the summary statistics from other consortia-based studies for the meta-analysis. We used GIANT [25] (*N* up to 327,665) for BMI, waist-to-hip ratio, and height; Global Lipids [26] (*N* up to 188,577) for lipid-related traits; MAGIC [27] (*N* up to 22,293) for diabetes-related traits; ICBP [29] (*N* = 697,307) for systolic and diastolic blood pressures, respectively; DIAGRAM [30] (*N* = 34,840 cases and 114,981 controls) for T2D; CARDIOGRAM [31] (*N* = 22,233 cases and 64,762 controls) for coronary artery disease (CAD); and EPIC-InterAct [32] (*N* = 12,722) for estimates of the *LCT* variant-milk intake association.

### Statistical analysis

Systolic (SBP) and diastolic (DBP) blood pressures were calculated by averaging 2 BP measurements, and for individuals taking BP-lowering medication in the 1958BC, HRS and the UK Biobank, we adjusted for the medication use by adding 15 and 10 mm Hg to SBP and DBP, respectively [28]. Pulse pressure (PP) was calculated as SBP minus DBP. For participants on lipid-lowering medication, we adjusted for their medication use by dividing their biomarker concentrations by a biomarker-specific correction factor (0.68 for LDL-C, 1.05 for HDL-C, 0.75 for total cholesterol, 0.87 for triglycerides, 1.21 for CRP, and 1.04 for HbA1c), which was obtained from the UK Biobank [33]. In the analysis related to HbA1c, we have also adjusted for anti-diabetic mediation use in the regression model. The primary data analyses in 417,236 individuals assumed an additive genetic model to be consistent with look-up results from consortia-based studies. However, we had also analysed the data under a recessive model, with participants bearing ‘CT’ and ‘TT’ combined and ‘CC’ homozygotes as the reference, wherever appropriate. Chi-square test was used to assess whether the genotype frequencies were in Hardy-Weinberg Equilibrium (1958BC, *P* = 0.522; HRS, *P* < 0.0001; UK Biobank, *P* = 0.088). The genetic associations with the continuous outcomes were examined using linear regression, with models adjusted for gender, BMI, assessment centre, SNP array and region of residence (1958BC) or principal components (HRS and UK Biobank) to account for population stratification. All cardio-metabolic traits [except SBP, DBP and glycated haemoglobin (HbA1c)] were inverse-normal-transformed to be consistent with the look-up results from consortia. The data from the 1958BC, HRS, the UK Biobank and consortia were meta-analysed using the random effects meta-analysis where estimates were available for at least two studies (Supplementary Table 1). To quantify the causal effects of milk intake, estimates of *LCT*-cardio-metabolic-traits associations from the meta-analysis were taken forward for the MR analysis, where the causal effect was estimated using the instrumental variable ratio method, with the estimate of *LCT*-milk intake association taken from the EPIC-interAct study [32]. For the instrument validation analysis, the *LCT* gene variant was tested for its association with age, sex, social or lifestyle factors such as annual income, education, health status, smoking and alcohol and coffee consumption (*P* value < 0.00625, Bonferroni corrected threshold considered statistically significant). We conducted all analyses using STATA, version 14.1 (StataCorp LP, College Station, Texas, USA), with MR analyses performed using the mrratio command in the mrrobust package [34].

To increase the statistical power, we used summary statistics from consortia-based studies to meta-analyze with the data from the three population-based studies. Results from consortia meta-analyses were publicly available, and in the GIANT [25], Global Lipids [26], MAGIC [27], ICBP [29], DIAGRAM [30] and CARDIOGRAM [31], each study had performed the association analyses assuming an additive genetic model.

## Results

### Association of the LCT gene variant with milk intake

Table 1 shows the frequency of milk consumption stratified based on gender, BMI and *LCT* gene variant. The odds of consuming milk was higher for the ‘T’ allele carriers of the *LCT* variant (SNP rs4988235) compared to the CC homozygotes (OR: 2.14, 95% CI: 1.57–2.93, *P* = 1.68 × 10^−6^ for the 1958BC; OR 1.21, 95% CI: 1.09–1.34, *P* = 3.0 × 10^−4^ for the UK Biobank). In the UK biobank, 1.83% of ‘TT’ carriers followed a lactose free diet compared to 1.77% and 2.40% in CT and CC carriers, respectively (*P* = 7.6 × 10^−5^). The observed patterns of milk consumption varied strongly by age, sex, social or lifestyle factors such as annual income, education, health status, smoking and alcohol and coffee consumption, while no notable imbalances were observed by *LCT* genotype (Supplementary Table 2). Furthermore, check against the GWAS catalogue (https://www.ebi.ac.uk/gwas/), confirmed the association between *LCT* rs4988235 and BMI (*P* = 2 × 10^−6^) [25], with no other pleiotropic associations reported.

**Table 1:** Frequency of milk consumption stratified based on gender, body mass index (BMI) and gene variant.

	1958 British Birth Cohort			UK Biobank			EPIC InterAct	
	Never drinkers (*N* = 1701)	Infrequent/daily drinkers (*N* = 3742)	*P* value	Non-dairy consumers (*N* = 31,731)	Milk drinkers (*N* = 374,202)	*P* value	Milk intake (g/day)^a^ (*N* = 12,722)	*P* value
Gender								
Men	846 (28.66%)	2106 (71.34%)		11,519 (6.17 %)	175,071 (93.83 %)		–	–
Women	855 (34.32%)	1636 (65.68%)	7.05 × 10^−^^06^	20,212 (9.21 %)	199,131 (90.79 %)	2.023 × 10^−283^	–	–
BMI								
Underweight	10 (37.04%)	17 (62.96%)		307 (15.16 %)	1718 (84.84 %)		–	–
Normal	571 (31.67%)	1232 (68.33%)		12,550 (9.54 %)	119,030 (90.46 %)		–	–
Overweight	702 (30.54%)	1597 (69.46%)		12,129 (7.01 %)	160,852 (92.99 %)		–	–
Obese	416 (31.78%)	893 (68.22%)	0.732	6612 (6.74 %)	91,450 (93.26 %)	9.073 × 10^−218^	–	–
*LCT* gene variant (additive model)								
TT	917 (30.29%)	2110 (69.71%)		17,287 (7.67 %)	207,963 (92.33 %)		188 (48–373)	
CT	657 (31.99%)	1397(68.01%)		11,108 (7.81 %)	131,163 (92.19 %)		160 (36–299)	
CC	127 (35.18%)	234 (64.82%)	0.111	2090 (9.12 %)	20,820 (90.88 %)	7.02 × 10^−14^	146 (19–225)	2.00 × 10^−7^
*LCT* gene variant (recessive model)								
TT + CT	1574 (30.98%)	3507 (69.02%)		28,395 (7.73 %)	339,126 (92.27 %)		–	
CC	127 (35.18%)	234 (64.82%)	0.096	2090 (9.12 %)	20,820 (90.88 %)	2.104 × 10^−14^	–	–

^a^Data are expressed as median (quartile 1–quartile 4).

### Phenotypic associations

In the 1958BC, there was no significant association between high milk intake and cardiometabolic traits (*P* > 0.09) (Table 2). In the UK Biobank, there was an association of high milk intake with higher BMI (*P* = 3.72 × 10^−124^), SBP (*P* = 1.06 × 10^−6^), DBP (*P* = 0.009), low-density lipoprotein cholesterol (LDL-C) (*P* = 1.20 × 10^−19^), TC (*P* = 2.75 × 10^−12^), triglycerides (*P* = 8.33 × 10^−19^), and C-reactive protein (CRP) (*P* = 2.90 × 10^−35^) and lower HDL-C (*P* = 8.31 × 10^−6^) and HbA1c (*P* = 7.96 × 10^−14^). Meta-analysis of the data from 1958BC and UK Biobank showed that there was an association of high milk intake with lower HDL-C (*P* = 2.70 × 10^−6^) and higher triglycerides (*P* = 0.001) and CRP (*P* = 2.60 × 10^−30^).

**Table 2:** Phenotypic and Genetic associations between milk intake and cardio-metabolic traits.

	The UK Biobank		1958BC			HRS		Consortia		Meta-analysis^a^		Heterogeneity		Weight			
	Beta ± SE (*N*)	*P* value	Beta ± SE (*N*)		*P* value	Beta ± SE (*N*)	*P* value	Beta ± SE (*N*)	*P* value	Beta ± SE^b^	*P* value	*P* value	I2 (%)	UK Biobank	1958BC	HRS	Consortia
BMI																	
Phenotypic	0.146 ± 0.006 (*N* = 404,648)	3.72E−124	−0.015 ± 0.029 (*N* = 5438)		0.61	NA	NA	NA	NA	0.068 ± 0.08	0.40	5.60E−08	96.6	51.55	48.45	–	–
Genetic	0.014 ± 0.003 (*N* = 375,247)	2.73E−07	0.0005 ± 0.021 (*N* = 5672)		0.98	−0.008 ± 0.018 (*N* = 8445)	0.65	0.016 ± 0.003 (*N* = 311,359)	4.60E−06	0.015 ± 0.002	4.68E−12	0.64	0	61.6	–	–	38.4
WC (adjusted for BMI)																	
Phenotypic	−0.000003 ± 0.004 (*N* = 404,571)	1.00	−0.016 ± 0.021 (*N* = 5424)		0.44	NA	NA	NA	NA	−0.0007 ± 0.004	0.88	0.45	0	95.99	4.01	–	–
Genetic	−0.001 ± 0.002 (*N* = 375,174)	0.51	−0.012 ± 0.015 (*N* = 5656)		0.42	0.011 ± 0.02 (*N* = 4684)	0.59	0.01 ± 0.004 (*N* = 223,634)	0.007	0.004 ± 0.006	0.49	0.007	86.2	53.81	–	–	46.19
SBP (mmHg)																	
Phenotypic	0.56 ± 0.11 (*N* = 369,133)	1.06E−06	−0.39 ± 0.45 (*N* = 5421)		0.38	NA	NA	NA	NA	0.18 ± 0.47	0.69	0.04	76.4	60.35	39.65	–	–
Genetic	−0.0058 ± 0.052 (*N* = 342,729)	0.91	0.28 ± 0.33 (*N* = 5654)		0.40	0.26 ± 0.66 (*N* = 4694)	0.70	0.008 ± 0.036^c^ (*N* = 697,305)	0.83	0.0085 ± 0.035	0.81	0.71	0	–		0.29	99.71
DBP (mmHg)																	
Phenotypic	0.17 ± 0.064 (*N* = 369,142)	9.29E−03	−0.45 ± 0.30 (*N* = 5421)		0.14	NA	NA	NA	NA	−0.071 ± 0.30	0.81	0.05	74.9	61.48	38.52	–	–
Genetic	0.040 ± 0.029 (*N* = 342,737)	0.17	0.18 ± 0.22 (*N* = 5654)		0.42	0.29 ± 0.39 (*N* = 4694)	0.47	0.025 ± 0.021^c^ (*N* = 697,307)	0.23	0.026 ± 0.021	0.21	0.51	0	–	–	0.28	99.72
HDL-C																	
Phenotypic	−0.024 ± 0.005 (*N* = 352,898)	8.31E−06	−0.051 ± 0.030 (*N* = 5334)		0.09	NA	NA	NA	NA	−0.025 ± 0.005	2.70E−06	0.37	0	96.8	3.2	–	–
Genetic	−0.015 ± 0.002 (*N* = 327,286)	3.01E−10	−0.037 ± 0.022 (*N* = 5563)		0.09	0.013 ± 0.029 (*N* = 3553)	0.66	−0.014 ± 0.004 (*N* = 183,570)	0.001	−0.015 ± 0.002	9.40E−13	0.59	0	71.38	–	0.5	28.12
LDL-C																	
Phenotypic	0.055 ± 0.006 (*N* = 384,881)	1.20E−19	−0.045 ± 0.030 (*N* = 5033)		0.13	NA	NA	NA	NA	0.009 ± 0.050	0.86	0.0009	90.85	54.21	54.21	–	–
Genetic	−0.023 ± 0.003 (*N* = 356,937)	5.25E−17	−0.030 ± 0.022 (*N* = 5247)		0.17	NA	NA	−0.028 ± 0.004 (*N* = 169,531)	3.20E−11	−0.024 ± 0.002	2.08E−26	0.32	0	70.38	–	–	29.62
TC																	
Phenotypic	0.042 ± 0.006 (*N* = 385,586)	2.76E−12	−0.054 ± 0.029 (*N* = 5341)		0.06	NA	NA	NA	NA	−0.002 ± 0.048	0.96	0.001	90.67	54.28	45.72	–	–
Genetic	−0.027 ± 0.003 (*N* = 357,601)	8.77E−24	−0.055 ± 0.021 (*N* = 5570)		0.009	0.003 ± 0.026 (*N* = 4228)	0.92	−0.031 ± 0.004 (*N* = 183,761)	4.00E−14	−0.028 ± 0.002	2.40E−36	0.38	0	67.99	–	0.72	31.3
Triglycerides																	
Phenotypic	0.049 ± 0.006 (*N* = 385,287)	8.30E−19	0.016 ± 0.026 (*N* = 5330)		0.53	NA	NA	NA	NA	0.043 ± 0.013	0.001	0.22	34.36	79.98	20.02	–	–
Genetic	0.001 ± 0.003 (*N* = 357,317)	0.56	−0.051 ± 0.019 (*N* = 5559)		0.008	NA	NA	−0.003 ± 0.004 (*N* = 174,267)	0.49	0.00007 ± 0.002	0.97	0.32	0.87	69.22	–	–	30.78
HbA1c (%)																	
Phenotypic	0.020 ± 0.003 (*N* = 385,644)	7.96E−14	0.006 ± 0.015 (*N* = 5339)		0.68	NA	NA	NA	NA	0.019 ± 0.003	1.10E−13	0.38	0	97.04	2.96	–	–
Genetic	−0.002 ± 0.001 (*N* = 357,767)	0.18	−0.002 ± 0.011 (*N* = 5568)		0.82	0.011 ± 0.017 (*N* = 4408)	0.54	−0.0001 ± 0.002 (*N* = 123,665)	0.96	−0.001 ± 0.001	0.27	0.61	0	67.66	–	0.38	31.95
CRP																	
Phenotypic	0.069 ± 0.006 (*N* = 384,759)	2.90E−35	0.042 ± 0.027 (*N* = 5276)		0.11	NA	NA	NA	NA	0.068 ± 0.006	2.60E−30	0.31	1.56	95.04	4.96	–	–
Genetic	0.002 ± 0.003 (*N* = 356,829)	0.53	0.03 ± 0.02 (*N* = 5503)		0.13	0.003 ± 0.027 (*N* = 4246)	0.91	NA	NA	0.002 ± 0.002	0.42	0.37	0	97.55	1.6	0.85	–

*NA* not available, *1958BC* 1958 British Birth Cohort, *HRS* the Health and Retirement study, *BMI* body mass index, *WC* waist circumference, *SBP* systolic blood pressure, *DBP* diastolic blood pressure, *CRP* C-reactive protein, *HDL-C* high density lipoprotein cholesterol, *LDL-C* low density lipoprotein cholesterol, *TC* total cholesterol.

^a^Random effects meta-analysis of the data from UK Biobank, the 1958BC, the HRS, and consortia-based studies was performed (after excluding the overlapping studies); Meta-analyses for BMI and WC have excluded 1958BC and HRS; meta-analyses for SBP and DBP have excluded 1958BC and UK Biobank; meta-analyses for HDL, LDL-C, TC, Triglycerides and HbA1c have excluded 1958BC.

^b^Random effect model was used for all meta-analyses.

^c^Results for the *LCT* variant were not available, therefore its proxy rs6754311 (*r*^2^ = 1 among British in England and Scotland) was used.

### Association of the LCT gene variant with cardio-metabolic traits

The ‘T’ allele indexing greater milk intake had a consistent association with higher BMI in the UK Biobank (*P* = 2.73 × 10^−7^), GIANT consortium (*P* = 0.001), and the meta-analysis (*P*_meta-analysis_ = 4.68 × 10^−12^) (Table 2). In contrast, *LCT* genotype was not associated with BMI adjusted waist circumference (WC) (indicator for central adiposity, *P*_meta-analysis_ = 0.49).

All individual studies with available data showed an inverse association between *LCT* genotype indexing greater milk intake and lower serum LDL-C (*P*_meta-analysis_ = 2.08 × 10^−26^), HDL-C (*P*_meta-analysis_ = 9.40 × 10^−13^), and total cholesterol concentrations (*P*_meta-analysis_ = 2.38 × 10^−36^) (Table 2). None of the genetic associations between the ‘T’ allele indexing higher milk intakes and SBP, DBP, and HbA1c were statistically significant (*P*_meta-analysis_ > 0.11). Information for CRP was only available for the 1958BC and UK Biobank (*P*_meta-analysis_ = 0.49).

Analyses in the UK Biobank confirmed the association between *LCT* variant and a higher total body fat percentage (*P* = 1.63 × 10^−7^), with a consistent association with higher fat-% noted across all indicators of regional fat distribution (Table 3). The estimates from these genetic associations were used in the IV ratio analysis, which confirmed the causal association of high milk intake with higher total body fat (*P* = 2.40 × 10^−4^), leg fat (*P* = 2.59 × 10^−4^), arm fat (*P* = 4.27 × 10^−4^) and trunk fat (*P* = 3.01 × 10^−4^).

**Table 3:** Milk intake and body fat distribution in the UK Biobank.

	*N*	Beta ± SE	*P* value
Total body fat			
Phenotypic	398,654	0.086 ± 0.005	8.38E−76
Genetic	369,696	0.011 ± 0.002	1.63E−07
IV ratio (per 50 g milk intake)	369,696	0.031 ± 0.009	2.40E−04
Leg fat			
Phenotypic	398,853	0.078 ± 0.004	1.59E−95
Genetic	369,879	0.009 ± 0.002	2.18E−07
IV ratio (per 50 g milk intake)	369,879	0.025 ± 0.007	2.59E−04
Arm fat			
Phenotypic	398,799	0.084 ± 0.005	1.46E−71
Genetic	369,834	0.010 ± 0.002	1.37E−06
IV ratio (per 50 g milk intake)	369,834	0.029 ± 0.008	4.27E−04
Trunk fat			
Phenotypic	398,639	0.095 ± 0.006	1.67E−64
Genetic	369,683	0.012 ± 0.002	3.92E−07
IV ratio (per 50 g milk intake)	369,683	0.036 ± 0.01	3.01E−04

In the UK Biobank, there was an association of the ‘T’ allele with T2D (*P* = 0.007), where ‘T’ allele carriers had 11% lower risk of diabetes; but the associations of *LCT* variant with CVD was not statistically significant (*P* = 0.302) (Table 4). However, in consortia look-up, there was no evidence for the association of *LCT* variant with T2D in the DIAGRAM consortium (*P* = 0.201) but there was an association with CAD in the CARDIoGRAM consortium (*P* = 0.030), where ‘T’ allele carriers had 14% lower risk of CAD.

**Table 4:** Association of milk intake with cardiovascular diseases and type 2 diabetes.

Phenotypes	*N*_cases_/*N*_controls_	OR^a^	95% CI	*P* value
DIAGRAM				
Type 2 diabetes	34,840/114,981	1.06	0.97–1.16	0.201
CARDIoGRAM				
Coronary artery disease	22,233/64,762	0.86	0.75–0.99	0.030
UK Biobank				
Type 2 diabetes	20,820/351,375	0.89	0.82–0.97	0.007
Cardiovascular disease	42,317/332,930	0.97	0.92–1.03	0.302

*CARDIoGRAM* Coronary ARtery DIsease Genome-wide Replication and Meta-analysis, *DIAGRAM* DIAbetes Genetics Replication And Meta-analysis, *OR* odds ratio, *CI* confidence intervals.

^a^Effects are presented as odds ratios and 95% confidence intervals, per 50 g milk intake.

**Fig. 1:**
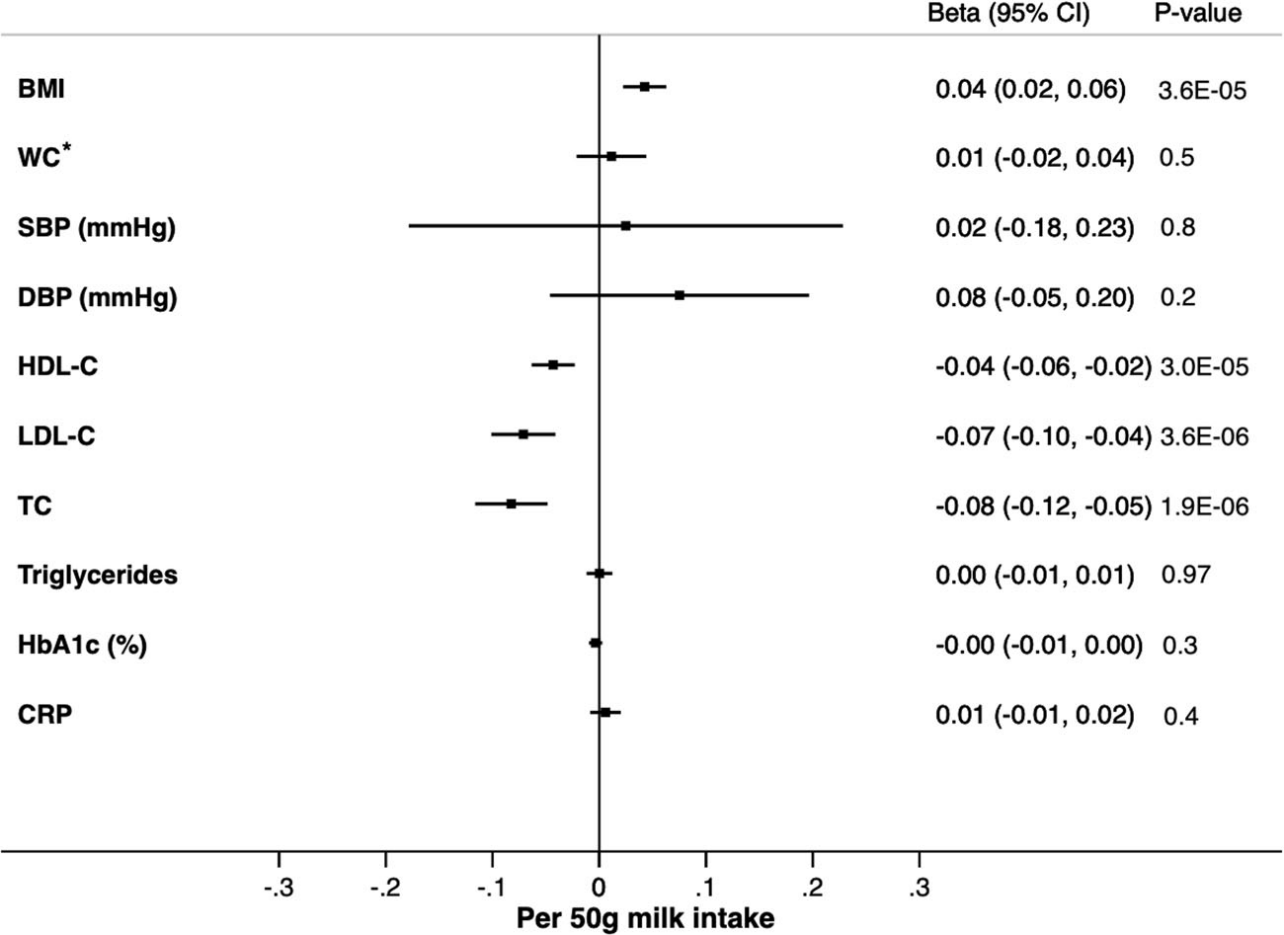
Mendelian Randomization (MR) estimates for the association between milk intake and cardio-metabolic traits. Using the estimates taken from the results of meta-analysis in Table 2 and estimates of the *LCT* variant-milk intake association from the EPIC-interAct study, the MR analysis showed a causal association of the genetically instrumented high milk intake with higher BMI (*P* = 3.60 × 10^−5^) and lower LDL-C (*P* = 3.60 × 10^−6^), total cholesterol (*P* = 1.90 × 10^−6^) and HDL-C (*P* = 3.00 × 10^−5^). Differences are shown as SD increase in the outcome with the exception of SBP (mmHg), DBP (mmHg), and HbA1c (%). * WC adjusted for BMI. SD standard deviation, *BMI* body mass index, SBP systolic blood pressure, DBP diastolic blood pressure, HDL-C high density lipoprotein cholesterol, LDL-C low density lipoprotein cholesterol, HbA1c glycated haemoglobin, CRP C-reactive protein, CI confidence interval.

Using the estimates taken from the results of meta-analysis in Table 2 and estimates of the *LCT* variant-milk intake association from the EPIC-interAct study (https://care.diabetesjournals.org/content/42/4/568.long), [32] the MR analysis confirmed the causal association of the genetically instrumented high milk intake with higher BMI (*P* = 3.60 × 10^−5^) and lower LDL-C (*P* = 3.60 × 10^−6^), total cholesterol (*P* = 1.90 × 10^−6^) and HDL-C (*P* = 3.00 × 10^−5^) (Fig. 1).

## Discussion

Our large-scale meta-analysis in up to 1,904,220 participants demonstrates that ‘T’ allele of the *LCT* SNP rs4988235 indexing greater milk intake is significantly associated with CVD-related risk factors such as higher BMI and lower TC and LDL-C concentrations. Furthermore, using a two-sample MR analysis, we confirmed the causality between genetically instrumented high milk intake and higher BMI and lower LDL-C, total cholesterol and HDL-C. In summary, our MR study represents an extensive exploration of the *LCT* gene variant for associations with milk intake and cardio-metabolic traits which is strengthened by the use of large datasets and consortia**-**based studies. Given the existing controversies between high milk intake and CVD risk, our MR analysis will have significant public health implications.

As an exposure, dairy milk is relatively complex, and in addition to calcium, dairy products constitute an important dietary source of protein and primarily saturated fat. Several intervention studies have investigated the association between dairy consumption and cardiovascular risk factors such as lipid outcomes [9, 35, 36]. However, most of the studies have been focused on comparing different types of milk (i.e. low fat vs. high fat), while there are only a few studies investigating the effects of milk per se. In addition, most of the studies were small with short duration and lacked adequate control groups, control of dietary composition and compliance measures. For instance, a crossover feeding study in eight men showed that the mean total and LDL-C were 7% and 11% lower, respectively, after 6 weeks consumption of a controlled low fat diet containing skim milk, compared to whole milk, suggesting that reducing milk fat consumption led to more favourable lipid profiles [9]. In addition, a few studies have also shown that skim milk appeared to have advantages on lowering cholesterol levels over whole milk or full cream milk [37, 38]; however, there was no effect on serum triglycerides [37]. On the other hand, studies that compared the effect of isoenergetic amounts of milk, cheese and butter on lipid outcomes showed that total cholesterol concentration was significantly lower after cheese than butter diet [35, 36]; however, a differential effect of fat in milk and butter was not confirmed [36]. In addition, a few prospective cohort studies have failed to show that higher intakes of milk and dairy products, regardless of milk fat levels, are associated with an increased risk of CVD-related outcomes. Hence, it is still not clear whether it is the fat content in dairy products that is contributing to the lower cholesterol levels or it is due to an unknown ‘milk factor’. Given the difficulties to assess the biological effects of dairy milk per se, we used the *LCT* gene variant (rs4988235) as an instrument to examine the causal association between milk intake and lipid outcomes, as genetic associations are less prone to confounding and free from reverse causation [39]. Our study using *LCT* gene variant as a genetic instrument confirmed the causal association between high milk consumption and lower TC and LDL-C in the combined meta-analysis. While the RCTs supplementing whole milk into the diet either lowered or failed to alter TC or LDL-C [37, 38, 40, 41], our large-scale study suggests an inverse association between high milk consumption and TC.

Some of the explanations for the lower cholesterol levels in response to high milk intake are as follows. Firstly, calcium and lactose in the milk have been shown to affect lipid metabolism where dietary lactose can affect lipaemia by enhancing calcium absorption and that the administration of a high amount of lactose results in decreased cholesterol content in humans [42]. Secondly, compared to other dairy products, milk is generally lower in fat and is also possible, that in order to increase their calcium intakes, individuals who are lactose intolerant tend to use more high fat dairy products such as cheese and many yogurts as these are generally better tolerated [43]. Thirdly, it is possible that dietary calcium intake may increase excretion of bile acids leading to the regeneration of bile acids from hepatic cholesterol and, eventually, resulting in the lowering of cholesterol concentrations [44]. Lastly, it could also be an outcome of gut microbial fermentation of indigestible carbohydrates, that are capable of altering cholesterol synthesis and disrupting enterohepatic circulation, thereby lowering cholesterol levels [45].

Several MR studies have been carried out examining the causal relationship between genetically instrumented milk intake and cardio-metabolic traits. While studies have shown associations of the *LCT* gene variant with obesity [46–49], abdominal obesity [50], diabetes [51], metabolic syndrome [46, 51] and their related traits such as BMI [46–49], WC [48], fat percentage [47] and fasting glucose [52] a few studies have failed to confirm these associations [16, 21, 50, 52, 53]. These inconsistencies could be because of the differences in the sample size, ethnicities, outcome measures and study design. To address some of these issues, we have used a large sample size (*N* = 417,236) from three population-based studies focussing on Caucasian populations from the UK and the US in a cross-sectional study design. In addition, we have used the summary statistics from consortia-based studies (*N* up to 1,077,312) and based our inferences on consistent association seen across the different large-scale studies included in our analyses. While the phenotypic associations showed a significant heterogeneity due to differences in the measurement of milk intake across the studies, there was no heterogeneity in the genetic associations, which suggests the significance of using MR studies to explore causal relationships.

The association between milk intake and BMI has been explored in several studies. The National Health and Nutrition Examination Survey (NHANES) in 1493 children (2–4 years) showed that milk had a more consistent positive association with BMI than other dairy products [7]. This finding was confirmed in the Early Childhood Longitudinal Survey in 8950 children which also showed that higher milk consumption was associated with higher *z*-scores of BMI at 4 years of age [54]. In contrast, a longitudinal study in 19,352 Swedish women (40–55 years) showed that milk consumption was inversely associated with weight gain, where participants who consumed at least one serving of milk per day had an odds ratio of 0.85 (95% CI: 0.73, 0.99) [55]. Similarly, a study in 1001 adolescents also showed an inverse association between milk intake and BMI [56]. Despite large samples, the findings have been inconsistent. However, genetic studies including a meta-analysis have shown that the ‘T’ allele carriers of the SNP rs4988235 (LP genotype) had higher BMI [51, 57], which is in line with our findings in the GIANT consortium (*N* = 327,665) and combined meta-analysis (*N* = 417,236), where the ‘T’ allele carriers had 0.02 and 0.015 kg/m^2^ higher BMI compared to the ‘CC’ homozygotes, respectively. Our study confirmed the causal association of the genetically instrumented milk intake with BMI and also body fat distribution (total body fat, leg fat, arm fat and trunk fat).

Despite some evidence from observational studies, and a smaller MR study [51] we did not find strong evidence for an association between milk intake and T2D or the regulation of long-term glucose metabolism indicated by HbA1c. A recent MR study in 21,820 European individuals has also failed to provide a genetic evidence for the association of milk intake with T2D [32]. Even though our study failed to show an association with T2D in the DIAGRAM consortium, we did find a significant association of the high milk intake ‘T’ allele with decreased T2D risk and lower HbA1c concentration in the UK Biobank cohort and with higher fasting insulin and HOMA-IR in the MAGIC consortium. These findings are in accordance with the study in 272 non-diabetic Caucasian women where those in the highest quartile of dairy consumption had higher HOMA-IR values than those in the lowest quartile [58]. In contrast, a prospective study in 2091 Chinese men and women showed that the dairy consumption was significantly associated with reduced risk of T2D [59]. In addition, a systematic review based on five studies that assessed the effect of dairy consumption on insulin sensitivity demonstrated that four studies showed a positive effect of dairy consumption on insulin sensitivity while one study failed to show any effect [60]. A recent study in 147,812 individuals from 21 countries has also shown higher intake of whole fat dairy was associated with lower incidence of diabetes [61]. These discrepancies could be because of the use of various dairy products rather than milk per se, small sample size and gender differences in sample selection. In contrast to our genetic associations, a study in 3575 Europeans failed to show an effect of the LP genotype with insulin level but showed an association with T2D [51]. Further large-scale meta-analyses including a broader range of diabetes-related traits may help to overcome these discrepancies in genetic associations.

In accordance with several randomised trials [5, 62, 63], our MR study also did not provide any evidence for a causal effect of high milk intake on CRP, an inflammatory biomarker. However, a few human intervention studies have shown a significant effect of milk consumption on other inflammatory biomarkers. An RCT in 58 Korean adults with metabolic syndrome investigated the effects of low-fat milk consumption on inflammatory and atherogenic biomarkers and found favourable changes for blood pressure and atherogenic markers, such as sVCAM‐1, sICAM‐1 and endothelin‐1, especially in those with hypertension or hypertriglyceridaemia [64]. Another intervention study in 42 smokers showed that non-supplemented milk (without fruit/vegetable extracts and vitamin C) significantly reduced inflammatory cytokines such as IL-6, IL-1beta and TNFA [65]. These favourable changes could be because the milk is rich in elements such as calcium, magnesium and potassium that help regulate blood pressure. In addition, casein and whey protein present in the milk are also considered as active biological peptides that inhibit angiotensin-I converting enzyme, an important factor in regulating blood pressure. Whether milk consumption exerts anti-inflammatory effects is presently questionable given the discrepancies in the findings from multiple studies and hence, further demonstration is required through well-designed and sufficiently powered studies.

One of the main strengths of our study is the large sample size. Besides using 1958BC (*N* = 5672), HRS (*N* = 8520) and the UK Biobank (*N* = 404,648), we have used the summary statistics from various consortia such as GIANT (*N* = 327,665), Global Lipids (*N* = 188,577), MAGIC (*N* = 22,293), ICBP (*N* up to 697,307), DIAGRAM (*N* = 34,840 cases and 114,981 controls) (T2D) and CARDIOGRAM (22,233 cases and 64,762 controls) to replicate our findings. Previous studies have shown conflicting findings regarding the association between high milk consumption and cardio-metabolic traits due to unknown confounding factors. However, our study has used the *LCT* gene variant as an instrument, which is unlikely to be affected by confounding factors, to establish the causal association. The use of large samples from the discovery cohorts, various consortia and genetic instrument has enabled us to make firm conclusions on the causal association between milk intake and cardio-metabolic traits such as BMI, BP, CAD, and lipids. Some of the limitations of the study include the lack of available information on gastro-intestinal diseases of the study participants and the type of milk (skim or whole) and the differences in the measurement of milk intake variable.

In conclusion, our study confirms the causal association between genetically instrumented high milk consumption and cardio-metabolic phenotypes, where the ‘T’ allele was associated with higher BMI and lower LDL-C and TC. Large intervention trials are needed to establish the causal link between high milk consumption and cardio-metabolic phenotypes before changes in dairy consumption could be recommended for the prevention of cardiometabolic traits.

## Supplementary information

Supplementary file
