# Supplementary material for: Evidence for a causal association between milk intake and cardiometabolic disease outcomes using a two-sample Mendelian Randomization analysis in up to 1,904,220 individuals
Source: Int J Obes (Lond). 2021 May 24;45(8):1751–62. doi: 10.1038/s41366-021-00841-2 (PMC8310799; doi:10.1038/s41366-021-00841-2)
Supplement: Supplementary file 1 — Supplementary file [file 41366_2021_841_MOESM1_ESM.docx]

**Supplementary file**

**Supplementary Table 1: Availability of the phenotypes in the study population and various consortia**

| **Phenotypes** | **1958BC** | **GIANT** | **GLGC** | **MAGIC** | **ICBP** | **DIAGRAM** | **CARDIo-GRAM** | **HRS** | **UK Biobank** | **Previously Published Studies** |
| --- | --- | --- | --- | --- | --- | --- | --- | --- | --- | --- |
| ***Milk intake*** | Y | - | - | - | - | - | - | - | Y | - |
| ***BMI*** | Y | Y | - | - | - | - | - | Y | Y | Y |
| ***WC*** | Y | **-** | - | - | - | - | - | Y | Y | - |
| ***WHR*** | Y | Y | - | - | - | - | - |  | Y | - |
| ***Height*** | Y | Y | - | - | - | - | - | Y | Y | - |
| ***SBP*** | Y | **-** | - | - | Y | - | - | Y | Y | Y |
| ***DBP*** | Y | **-** | - | - | Y | - | - | Y | Y | Y |
| ***CRP*** | Y | **-** | - | - | - | - | - | Y | Y | - |
| ***HDL-C*** | Y | **-** | Y | - | - | - | - | Y | Y | - |
| ***LDL-C*** | Y | **-** | Y | - | - | - | - | - | Y | - |
| ***TC*** | Y | **-** | Y | - | - | - | - | Y | Y | - |
| ***TGL*** | Y | **-** | Y | - | - | - | - | - | Y | - |
| ***HbA1c*** | Y | **-** | - | Y | - | - | - | Y | - | - |
| ***T2D*** | - | - | - | - | - | Y | - | - | - | - |
| ***CAD*** | - | - | - | - | - | - | Y | - | - | - |

1958BC: 1958 British Birth Cohort; HRS: the Health and Retirement study; GLGC: Global Lipids Genetics Consortium; GIANT: Genetic Investigation of Anthropometric Traits; MAGIC: the Meta-Analyses of Glucose and Insulin-related traits Consortium; ICBP: International consortium for Blood Pressure; Y: Data available; BMI, Body Mass Index; WC, Waist Circumference; WHR, Waist-Hip ratio; SBP, Systolic Blood Pressure; DBP, Diastolic Blood Pressure; CRP, C-Reactive Protein; HDL-C, High Density Lipoprotein Cholesterol; LDL-C, Low Density Lipoprotein Cholesterol; TC, Total Cholesterol; TGL, Triglycerides; T2D, Type 2 diabetes; CAD, Coronary Artery Disease.

**Supplementary table 2: Association of the *LCT* gene variant with age, sex, social and lifestyle factors**

| ***LCT genotypes*** | **Male**  **(%)** | **Age, yrs**  **Mean (SD)** | **High Income^*^**  **(%)** | **High Education^**^**  **(%)** | **Excellent Health**  **(%)** | **Smokers**  **(%)** | **Daily alcohol**  **drinkers (%)** | **Coffee, cups/day**  **Mean (SD)** |
| --- | --- | --- | --- | --- | --- | --- | --- | --- |
| CC | 46.17 | 56.97 (8.01) | 26.03 | 47.39 | 16.71 | 9.69 | 22.21 | 2.01 (2.20) |
| CT+TT | 45.92 | 56.91 (7.99) | 25.44 | 46.13 | 16.67 | 10.17 | 21.13 | 2.04 (2.13) |
| *P^#^_adj_* | *0.651* | *0.836* | *0.568* | *0.049* | *0.855* | *0.607* | *0.017* | *0.0043* |

^**^High education = Degree/professional; ^*^High income = ≥ £52,000 /year

^#^P values from the adjusted models with age, sex, SNP array, assessment centre and top 15 PCs as covariates

*Note: Bonferroni corrected type 1 error = 0.05/8 = 0.00625, meaning P values less than 0.00625 are considered statistically significant.*

**Study populations**

**The 1958 British birth cohort:** Detailed description of the 1958 British birth cohort (1958BC) has been published previously ^1^. In brief, study participants were born in England, Scotland or Wales during one week in March 1958 (n = 17,638). At age 45 years, 11,971 participants were invited to attend a biomedical survey: 9,377 (78%) completed at least one questionnaire. The 1958BC is almost entirely a white European population (98%)^2^, and for these analyses, 158 individuals of other ethnic groups and one pregnant participant were excluded. The 45-year biomedical survey was approved by the South-East Multi-Centre Research Ethics Committee (ref. 01/1/44), the ethics approval for genetic work was granted by the Joint UCL/UCLH Committees on the Ethics of Human Research (Committee A) Ref: 08/H0714/40, and written consent [for use of information in medical research studies] was obtained from the participants. For the present study, all the analyses were performed in up to 5,672 individuals.

*Anthropometric and clinical measurements*: Weight and standing height, at 45 years of age, were measured without shoes and in light clothing by a trained nurse using standardized protocols and equipment; waist circumference (WC) was measured by the nurse midway between the costal margin and iliac crest. BMI was calculated as weight (kg)/height (m)^2^. Blood pressure was measured in a seated position, after 5 min rest, using an Omron 705CP automated sphygmomanometer with a large cuff for participants with a mid-upper arm circumference ≥32 cm; the measurement was repeated three times, and blood pressure was determined as the average of successful measurements.

*Biochemical measurements*: Venous blood samples were drawn without prior fasting and posted to the collaborating laboratory. High-density lipoprotein cholesterol (HDL-c) and triglycerides were measured by standard auto-analyzer methodology. To allow for treatment effects amongst those taking lipid medications (n = 74), correction factors derived from the UK Biobank was used for the biomarkers to maintain consistency across the three cohorts. The details of the correction factors are included in the statistical analysis section in the main text. Low-density lipoprotein cholesterol (LDL-c) was calculated using the Friedewald formula ^3^. C-reactive protein (CRP) was assayed by immunonephelometry (Dade Behring, Milton Keynes, United Kingdom).

*Milk intake*: The milk intake variable was derived from the 45 yrs biomedical survey and based on the question how often they drink milk alone or in milky drinks ^4^. The responses were categorized based on the milk consumption question in the 1958BC: ‘*How often do you drink milk alone or in milky drinks such as hot chocolate, Horlicks, Complan*?’ 1) more than 4 times a day; 2) 2-4 times a day; 3) once a day; 4) 3-6 days a week; 5) 1 or 2 days a week; 6) less than 1 day a week; 7) occasionally; 8) never. In our analysis, infrequent/daily milk drinkers were those who chose an answer from 1 - 7, and never drinkers were those who selected the answer 8.

*SNP Genotyping*: Genome-wide data for the 1958BC were obtained through two sub-studies, both using the 1958BC participants as population controls. The first sub-study included 3000 DNA samples randomly selected as part of the Welcome Trust Case Control Consortium (WTCCC2) and genotyped on the Affymetrix 6.0 platform ^5^. The second sub-study was the Type 1 diabetes case-control study (T1DGC) which used 2,500 DNA samples and genotyped using the Illumina Infinium 550K chip through the JDRF/WT Diabetes and Inflammation Laboratory (DIL) ^6^. Region or residence was included in genetic models and coded as Scotland, North of England, Middle of England including Wales, and South of England.

**Health and Retirement study (HRS):** The HRS is a nationally representative longitudinal survey of more than 37,000 individuals in 23,000 households over age 50 in the United States ^7^. Data for this study comes from older Americans with European ancestry who participated in the 2006 wave of the US HRS. All participants provided informed consent to participate. HRS was approved by the University of Michigan Health Sciences Human Subjects Committee and this analysis was determined exempt by the Harvard School of Public Health Office of Human Research Administration. The HRS did not have information on milk intake and hence the data on up to 8,520 participants was used to investigate the association of the high milk intake allele (‘T’) with BMI, WC, HDL-C, TC, HbA1c, blood pressure and CRP. The correction for lipid- and BP- lowering medications was applied to the clinical and biochemical markers using the methods outlined in the statistical analysis section in the main text.

*Genotyping*: The samples were genotyped in two phases. The first phase consisted of DNA from buccal swabs collected in 2006 and extracted using the Qiagen Autopure method. The second phase consisted of saliva samples collected in 2008 and extracted with Oragene. Although the two phases were genotyped separately, the raw data were clustered and called together. The samples were genotyped in batches corresponding to 96-well plates. Additionally, 30 Oragene duplicates were genotyped with the buccal samples, and 30 buccal duplicates were genotyped with the Oragene samples. The DNA samples were genotyped at the Center for Inherited Disease Research (CIDR) using the Illumina HumanOmni2.5-4v1 array and using the calling algorithm GenomeStudio version 2011.2, Genotyping Module 1.9.4 and GenTrain version 1.0. The SNP annotation used by CIDR was “HumanOmni2.5-4v1 D”, but during data cleaning at the Genetics Coordinating Center of the University of Washington (UWGCC), the annotation was updated to “HumanOmni2.5-4v1 H.”

**The UK Biobank:** The UK Biobank is a major national and international health resource (http://www.ukbiobank.ac.uk), and a registered charity in its own right, with the aim of improving the prevention, diagnosis and treatment of a wide range of serious and life-threatening illnesses. The UK Biobank comprises ∼500,000 community-dwelling participants who were initially recruited from across Great Britain between 2006 and 2010, aged 40–69 years ^8^. For the present study, we used up to 404,648 individuals with information on *LCT* gene variant, milk intake and outcomes relating to obesity, and blood pressure. We have restricted the analyses to individuals genetically defined as Caucasian and to persons that are unrelated. The present analyses were conducted under UK Biobank application number 20175. All participants provided informed consent to participate. Further information on the consent procedure can be found here (<http://biobank.ctsu.ox.ac.uk/crystal/field.cgi?id=200>). Details on the laboratory procedures for biomarker measurements are available at <http://www.ukbiobank.ac.uk/wp-content/uploads/2013/11/BCM023_ukb_biomarker_panel_website_v1.0-Aug-2015.pdf>

The correction for medication use was applied to the biomarkers using the methods^9,10^ outlined in the statistical analysis section in the main text.

*Ascertainment of cardiovascular diseases*:

| **CVD** | **Source** | **Code** |
| --- | --- | --- |
| **CAD** | HES (ICD10) | I20, I21, I22, I23, I24, I25 |
|  | HES (ICD9) | 410, 411, 412, 413, 414, 415.1, 428, 429.2, 429.5, 429.6, 429.7 |
|  | HES (OPCs) | K40, K41, K42, K43, K44, K45, K46, K47.1, K49, K50, K59, K75, X50 |
|  | Baseline (non-cancer illness code) | 1074, 1075 |
|  | Baseline (operation) | 1095, 1070 |
|  | Cause of death (ICD10 primary + secondary) | I20, I21, I22, I23, I24, I25 |
| **PAD** | HES (ICD10) | I73.1, I73.8, I73.9 |
|  | HES (ICD9) | 443.1, 443.8, 443.9 |
|  | HES (OPCs) | L50, L51, L52, L53, L54, L58, L59, L60, L62, L63, L65 |
|  | Baseline (non-cancer illness code) | 1067, 1087, 1492, 1591 |
|  | Baseline (operation) | 1071, 1102, 1103, 1104, 1107, 1108, 1515 |
|  | Cause of death (ICD10 primary + secondary) | I73.1, I73.8, I73.9 |
| **Stroke** | HES (ICD10) | G46.3, G46.4, G46.5, G46.6, G46.7, I60, I61, I62.0, I62.1, I62.9, I63, I64, I69.0, I69.1, I69.2, I69.3, I69.4, I69.8 |
|  | HES (ICD9) | 430, 431, 432.0, 432.1, 432.9, 433, 434, 436, 437.0, 437.9 |
|  | HES (OPCs) | U54.3 |
|  | Baseline: Vascular problem code | 3 |
|  | Baseline: Non cancer illness code | 1081, 1082, 1086, 1491, 1583 |
|  | Cause of death ICD10 (Primary + Secondary) | G46.3, G46.4, G46.5, G46.6, G46.7, I60, I61, I62.0, I62.1, I62.9, I63, I64, I69.0, I69.1, I69.2, I69.3, I69.4, I69.8 |

CVD, Cardiovascular disease; CAD, Coronary artery diseases; PAD, peripheral artery diseases; OPCS, OPCS Classification of Interventions and Procedures version 4; HES, Hospital Episodes Statistics; ICD, International Classification of Disease.

Type 2 diabetes status was ascertained from the first occurrences variable, n_130709_0_0 (ICD10 = E11). Among cases and controls, we also removed individuals with type 1 diabetes, who were identified using n_130707_0_0 (ICD10 = E10).

*Milk intake*: Milk information was derived from the question in the touchscreen questionnaire (<http://biobank.ctsu.ox.ac.uk/crystal/refer.cgi?id=100312>). The responses were categorized based on the milk consumption question in UK Biobank: ‘*What type of milk do you mainly use?*’ 1) full cream; 2) semi-skimmed; 3) skimmed; 4) soya; 5) other type of milk; 6) never/rarely have milk. In our analysis, milk drinkers were those who chose an answer from 1 - 3, and non-dairy consumers were those who selected the answer from 4 – 6 (**Table 1**).

*Genotyping*: Samples were genotyped at the Affymetrix Research Services Laboratory in Santa Clara, California, USA. Axiom Array plates were processed on the Affymetrix GeneTitan® Multi-Channel (MC) Instrument. Genotypes were then called from the resulting intensities in batches of ~4,700 samples (~4,800 including the controls) using the Affymetrix Power Tools software and the Affymetrix Best Practices Workflow. After genotype calling, Affymetrix performed quality control in each batch separately, to exclude SNPs with poor cluster properties. If a SNP did not meet the Affymetrix prescribed QC thresholds in a given batch, it was set to missing in all individuals from that batch. Affymetrix also checked sample quality (such as DNA concentration) and genotype calls were provided only for samples with sufficient DNA metrics. More information about the Affymetrix calling algorithms and quality control protocols were previously published.^11^

**European Prospective Investigation into Cancer and Nutrition (****EPIC)-InterAct:** EPIC-InterAct is a prospective case-cohort study nested within eight European countries of the EPIC study. From 340,234 adults of the EPIC study for whom baseline blood samples were available, EPIC-InterAct randomly selected a sub-cohort of 16,154 participants and identified 12,403 incident cases of type 2 diabetes between 1991 and 2007, including 778 cases from the sub-cohort by design. For the present analysis, we used the summary statistics from the study by Vissers et al^12^ (N=12,722).

**References**

1. Power C, Elliott J. Cohort profile: 1958 British birth cohort (National Child Development Study). *International journal of epidemiology* 2006; **35**(1): 34-41.

2. Atherton K, Fuller E, Shepherd P, Strachan DP, Power C. Loss and representativeness in a biomedical survey at age 45 years: 1958 British birth cohort. *J Epidemiol Community Health* 2008; **62**(3): 216-23.

3. Friedewald WT, Levy RI, Fredrickson DS. Estimation of the concentration of low-density lipoprotein cholesterol in plasma, without use of the preparative ultracentrifuge. *Clin Chem* 1972; **18**(6): 499-502.

4. Matthews H. Learning from Learners. *Educational Developments* 2004; **5**(3): 5-7.

5. WTCCC. Genome-wide association study of 14,000 cases of seven common diseases and 3,000 shared controls. *Nature* 2007; **447**(7145): 661-78.

6. Barrett JC, Clayton DG, Concannon P, et al. Genome-wide association study and meta-analysis find that over 40 loci affect risk of type 1 diabetes. *Nat Genet* 2009; **41**(6): 703-7.

7. Sonnega A, Faul JD, Ofstedal MB, Langa KM, Phillips JW, Weir DR. Cohort Profile: the Health and Retirement Study (HRS). *International journal of epidemiology* 2014; **43**(2): 576-85.

8. Astle WJ, Elding H, Jiang T, et al. The Allelic Landscape of Human Blood Cell Trait Variation and Links to Common Complex Disease. *Cell* 2016; **167**(5): 1415-29 e19.

9. Sinnott-Armstrong N, Tanigawa Y, Amar D, et al. Genetics of 35 blood and urine biomarkers in the UK Biobank. *Nature genetics* 2021; **53**(2): 185-94.

10. International Consortium for Blood Pressure Genome-Wide Association S, Ehret GB, Munroe PB, et al. Genetic variants in novel pathways influence blood pressure and cardiovascular disease risk. *Nature* 2011; **478**(7367): 103-9.

11. Affymetrix. “UKB_WCSGAX: UK Biobank 500K Samples Processing by the Affymetrix Research Services Laboratory”. 2015.

12. Vissers LET, Sluijs I, van der Schouw YT, et al. Dairy Product Intake and Risk of Type 2 Diabetes in EPIC-InterAct: A Mendelian Randomization Study. *Diabetes care* 2019; **42**(4): 568-75.
